# METACOGNITION AND EMOTIONS IN OLDER AFRICAN AMERICAN ADULTS

**DOI:** 10.1093/geroni/igac059.2069

**Published:** 2022-12-20

**Authors:** Katherine Kero, Colt M Halter, John L Woodard, Bruno Giordani, Ana Daugherty, Voyko Kavcic

**Affiliations:** Wayne State University, Detroit, Michigan, United States; Wayne State University, Detroit, Michigan, United States; Wayne State University, Detroit, Michigan, United States; University of Michigan, Ann Arbor, Michigan, United States; Wayne State University, Detroit, Michigan, United States; Wayne State University, Detroit, Michigan, United States

## Abstract

Older adults in the earliest stages of cognitive decline often present with subjective cognitive complaints which may not be fully reflected in objective measures of cognition. Previous research suggests that a relationship exists between negative emotions, stress and metacognition, but these relationships have not yet been examined in the context of COVID-19. The purpose of this study was to examine the role of stress and emotions in perceived cognition in the context of the COVID-19 pandemic. Telephone screenings were administered to 206 older African Americans (aged 64–94 years). Objective cognition (Telephone Interview for Cognitive Status [TICS]), subjective cognition (Cognitive Change Questionnaire [CCQ]), perceived stress scale 4 (PSS-4), and survey questions about affective responses to COVID-19 experiences were measured. Objective TICS scores predicted subjective CCQ executive function scores (F(1, 197)=4.37, p=.038, R2=.022). Discrepancy scores were calculated as the standardized residual variance between objective and subjective measures. Survey items describing emotional states were summarized with emodiversity scores following Quoidbach and colleagues’ (2014) formula. Discrepancy scores were correlated with perceived stress, as well as global and negative emodiversity (Spearman r=.294, .279, .318, p<.001). In conclusion, we have shown that objective and subjective measures of cognition are related, but discrepancies exist between objectively-measured and self-perceived cognition. Increased stress and greater negative emotions are associated with greater overestimation of cognitive difficulties relative to one’s objective level of cognition. As stress and negative emotions have increased for many during the pandemic, individuals may also have depreciated their self-appraisal of cognitive abilities in the present climate.

